# The association between one-year fall history and quality of life among older adults in the Geelong Osteoporosis Study: does fall frequency matter?

**DOI:** 10.1007/s41999-025-01324-7

**Published:** 2025-10-06

**Authors:** Tewodros Yosef, Julie A. Pasco, Monica C. Tembo, Kara L. Holloway-Kew

**Affiliations:** 1 Deakin University, Institute for Mental and Physical Health and Clinical Translation (IMPACT), School of Medicine – Barwon Health, Geelong, VIC Australia; 2 School of Public Health, College of Medicine and Health Sciences, Mizan-Tepi University, Mizan Teferi, Ethiopia; 3 Department of Medicine – Western Health, The University of Melbourne, St Albans, VIC Australia; 4 Department of Epidemiology and Preventive Medicine, Monash University, Melbourne, VIC Australia

**Keywords:** Falls, Geelong Osteoporosis Study, Quality of life, Ageing, WHOQOL-BREF

## Abstract

**Purpose:**

Older adults are at increased risk of falls, which can substantially impact their quality of life (QoL). While few global studies have explored this association, comprehensive research in Australia remains limited. This study aimed to assess the association between a one-year fall history and QoL domains and compare QoL between single and recurrent fallers in older Australians.

**Methods:**

Participants (n = 530, age ≥ 65 yr) were drawn from the 15-year assessment wave of the Geelong Osteoporosis Study. QoL was assessed using the World Health Organization Quality of Life-BREF (WHOQOL-BREF), a 26-item questionnaire covering four domains: physical, psychological, social, and environmental health. Falls within the previous 12 months were self-reported. Tobit regression was used to analyse associations between falls and QoL. Post-estimation linear combination tests were used to assess whether the associations differed between single and recurrent falls.

**Results:**

The participants had a mean age of 75.4 ± 7.2 years, and 266 (50.2%) were male. Recurrent falls were associated with all domains: physical (β = −5.94, 95% CI −10.8, −1.22), psychological (β = −7.92, 95% CI −13.7, −2.12), social (β = −10.7, 95% CI −18.4, −3.02), and environmental (β = −5.79, 95% CI −11.3, −0.32). Single falls were mainly associated with physical (β = −2.90, 95% CI −5.48, −0.32) and psychological (β = −3.20, 95% CI −5.59, −0.80) domains. Linear combination tests showed differences only in social and environmental domains.

**Conclusions:**

Recurrent falls are strongly associated with reduced QoL across all domains. Preventive strategies should prioritise older adults with recurrent falls to improve overall well-being and living conditions.

**Supplementary Information:**

The online version contains supplementary material available at 10.1007/s41999-025-01324-7.

## Key summary points

**Aim:**

To assess the association between a one-year fall history and QoL domains and compare QoL between single and recurrent fallers in older Australians.

**Findings:**

Recurrent falls were affected all QoL domains, while single falls were linked mainly to physical and psychological health. Compared to single fallers, those with recurrent falls experienced greater declines in social and environmental QoL domains.

**Message:**

Falls, particularly recurrent ones, diminish QoL in older Australians, highlighting the need for targeted prevention efforts and offering valuable guidance for promoting healthy ageing.

**Supplementary Information:**

The online version contains supplementary material available at 10.1007/s41999-025-01324-7.

## Introduction

As individuals age, falls become a major health concern [1, 2], which can greatly influence the quality of life (QoL) [3]. The growing number of older people and increased life expectancy are leading to more falls and fall-related injuries [4, 5]. Age is a key, non-modifiable factor influencing QoL, as it leads to health issues like chronic diseases, polypharmacy, sensory impairments, cognitive decline, and reduced physical and mental functioning, increasing falls [6–9].

The World Health Organization defines QoL as an individual’s perceptions of their life concerning cultural values, goals, and expectations [10]. QoL is a key indicator of health in older adults [7] and reflects physical, psychological, social, and environmental well-being [11]. It is shaped by a range of factors [6], including sociodemographic (e.g., age, gender, education), economic (e.g., income, employment) [6, 9], health-related (e.g., physical and mental health) [12], and psychosocial factors (e.g., social support, life satisfaction) [13]. Additionally, behavioural aspects such as poor sleep [14], smoking [6], physical inactivity, and eating problems [8], as well as nutritional issues like being underweight, overweight [15], or poorly nourished [16], are known to negatively affect QoL.

The relationship between falls and QoL is intricate. Typically, individuals who experience falls report a lower QoL compared to those who do not [17]. Thiem et al. [18] showed that self-reported falls over the previous 12 months were associated with a lower QoL. A larger number of falls over 36 months was associated with a greater decline in QoL [19]. The negative influence of falls is compounded by other underlying factors that influence both the occurrence of falls and overall QoL. Several factors influence both the likelihood of falls and QoL, including fall-related injuries (like fractures) [9], reduced physical function, fear of falling [20], and anxiety [8].

Social isolation or inadequate support networks can hinder recovery and negatively influence overall QoL [7, 21]. In some settings, financial constraints, such as being uninsured, can limit access to healthcare and fall prevention resources, negatively influencing QoL [22]. Although few studies have examined the association between falls and QoL among older adults globally [9, 17, 18, 20], domain-specific impacts, especially distinguishing between single and recurrent falls, remain underexplored. To our knowledge, this is the first study to examine the relationship between falls and domains of WHOQOL-BREF-rated QoL—physical, psychological, social, and environmental—among community-dwelling older adults in Australia. Therefore, this study aimed to assess the association between a one-year fall history and QoL domains and compare QoL between single and recurrent fallers in older Australians.

## Methods

### Study sample

This study is a secondary analysis of data from the Geelong Osteoporosis Study (GOS) [23], a cohort of residents from the Barwon Statistical Division in south-eastern Australia. The study recruited participants using a random sampling procedure from the Australian electoral roll, ensuring an even distribution of participants across the adult age range. A cohort of 1494 women aged 20–94 years was recruited between 1993 and 1997, with follow-up assessment waves conducted at 2, 4, 6-, 8-, 10-, and 15-years post-baseline. Recruitment for men occurred approximately a decade later, between 2001 and 2006, enrolling 1540 participants aged 20–92 years, with follow-up assessments conducted at 5 and 15 years. This cross-sectional analysis used data collected during the 15-year assessment wave for women (2011–2014) and men (2016–2022), as these represent the most recent complete assessments available at the time of writing, providing the most comprehensive information on falls, quality of life, and relevant covariates. The data were collected cross-sectionally from a single wave of a larger longitudinal study, rather than representing a continuous 15-year follow-up. Among the 1432 participants who completed the 15-year assessment wave, 530 were aged 65 years and older. Given our aim to investigate the association between falls and quality of life in older adults, the analysis was restricted to this group. Therefore, the final sample comprised all 530 eligible participants aged ≥ 65 years.

### Study variables

#### Dependent variable

The Australian version of the WHOQOL-BREF, a 26-item shortened form of the WHOQOL-100, was used to assess QoL [24]. The WHOQOL-BREF is a 26-item instrument that encompasses four domains: physical health (7 items), psychological health (6 items), social relationships (3 items), and environmental health (8 items). It also includes two global items that assess perceived self-rated QoL and general health satisfaction. Global quality of life (GQoL) represents a combined measure of perceived self-rated QoL and general health satisfaction. The physical health domain, consisting of seven items, covers aspects such as daily activities, reliance on medication, energy levels, mobility, pain, sleep, and work capacity. The psychological domain includes six items, assessing body image, negative and positive emotions, self-esteem, spirituality, and cognitive functioning. The social relationships domain, with three items, examines personal relationships, social support, and sexual life. Finally, the environment domain, made up of eight items, addresses financial stability, safety, access to health and social services, home environment, opportunities for skill and knowledge acquisition, recreation, transportation, and the physical environment (e.g., pollution, noise, traffic, climate). The GQoL and the four domains, with items measured on a 5-point Likert scale. These variables were treated and analysed as continuous variables due to having four or more levels [25]. The scores for each domain of the WHOQOL-BREF were calculated by summing the respective item scores for each respondent. These scores were then linearly transformed to a 0–100 scale, as recommended by the WHOQOL group. Higher domain scores reflect better QoL, as the scores are scaled positively [26]. Each WHOQOL-BREF domain demonstrated satisfactory reliability (Cronbach’s alpha > 0.7), as reported by West et al. [26].

#### Other measures

Sociodemographic, lifestyle, medication use, and fall data were collected through self-report. Educational attainment was categorised into four groups: never completed secondary school, completed secondary school, completed a Technical and Further Education (TAFE) or trade qualification, and completed a university degree. Marital status was grouped into three categories: single/never married, in a relationship/married, and separated/widowed. Sleep duration (in hours) was collected.

A fall was defined as an unexpected descent to the ground from a lying, sitting, or standing position [23]. Participants were asked if they had fallen in the past 12 months, with recurrent falls defined as ≥ 2 falls during this period. Smoking was self-reported, defined as regular use of cigarettes, cigars, or pipes. High alcohol consumption was defined as more than 2 standard drinks per day for women and 3 standard drinks per day for men, with each standard drink equal to 10 g of pure alcohol.

Mobility was assessed using a 7-item scale and categorised as high (very active and active) or low (sedentary to bedfast). Participants' use of mobility assistive devices (e.g., walkers or canes) was recorded (yes/no). Polypharmacy was defined as the use of five or more medications daily [27]. Comorbidities were assessed using the Charlson Comorbidity Index [28]. Symptoms of depression and anxiety were evaluated using the 7-item Hospital Anxiety and Depression Scale (HADS), rated on a 4-point Likert scale. Scores ranged from 0 to 21, with participants categorized as normal (0–7) or cases (8 +) [29].

Social support was assessed using the 12-item Multidimensional Scale of Perceived Social Support (MSPSS), rated on a 7-point Likert scale from 1 (very strongly disagree) to 7 (very strongly agree). The total score, ranging from 12 to 84, was used to categorize participants into low (12–35), medium (36–60), or high (61–84) perceived support [30]. Weight was measured using electronic scales (accurate to 0.1 kg), height with a Harpenden stadiometer (accurate to 0.001 m), and BMI was calculated by dividing weight by the square of height (kg/m^2^).

### Statistical analysis

The 15-year assessment wave data for men and women were combined due to the availability of similar outcome and exposure variables in both datasets, thereby increasing the sample size and enhancing statistical power. Continuous variables with a normal distribution were summarised as mean and standard deviation (SD), while those not normally distributed were described using the median and interquartile range (IQR). Categorical variables were summarised as frequencies and percentages. For group comparisons based on fall frequency, continuous variables were analysed using one-way ANOVA (e.g., QoL) when normality assumptions were met; otherwise, the Kruskal–Wallis test was applied (e.g., age, BMI, and MSPSS score). For variables with statistically significant results, we performed appropriate post hoc pairwise comparisons to identify specific group differences. Chi-square and Fisher’s exact tests were used to analyse associations between categorical variables. The linear relationships between the QoL domains, global QoL, and overall QoL were examined using Pearson correlation coefficients. Correlation coefficients are categorised as negligible (0.00–0.10), weak (0.10–0.39), moderate (0.40–0.69), strong (0.70–0.89), and very strong (0.90–1.00) [31]. Multicollinearity was assessed, and the variance inflation factor (VIF) was acceptable, being less than 2 [32].

Tobit regression is commonly applied when the dependent variable is censored, meaning it has a fixed lower or upper boundary [33]. For QoL domains, if scores are restricted within a range (such as 0 to 100), Tobit regression is suitable, as it accounts for the fact that scores cannot go beyond these limits. Falls were coded as a categorical variable: 0 for no falls, 1 for a single fall, and 2 for recurrent falls, allowing analysis across varying levels of fall risk. The association of falls and the physical health, psychological health, social relationships, and environmental health domains of QoL, as well as the overall QoL score, was assessed using Tobit regression analysis. The relationship between each covariate and the dependent variable was first assessed using crude (bivariable) analyses. This preliminary step aimed to identify candidate variables for inclusion in the multivariable regression model. Variables with a p-value less than 0.25 in the bivariable analysis [34] were considered for inclusion. This threshold helps to retain potentially important predictors while minimizing the risk of overfitting [35]. The multivariable model was adjusted for age, sex, education, marital status, body mass index, smoking, alcohol consumption, nighttime sleep duration, mobility, use of mobility assistive devices, social support, anxiety, depression, polypharmacy, decreased vision, and comorbidity index. Adjusted β coefficients with their 95% confidence intervals are reported. All p-values were two-tailed, with statistical significance set at p < 0.05. Pairwise comparisons of adjusted QoL scores across fall frequency categories were performed post-Tobit regression. To examine whether the beta coefficients for single and recurrent falls significantly differed within each model, we conducted post-estimation contrast tests using the linear combination (lincom) test. Data analysis was completed using Stata version 18 (StataCorp. 2023. Stata Statistical Software: Release 18. College Station, TX: StataCorp LLC).

## Results

### Participants characteristics

Table 1 shows the descriptive characteristics of the participants. Of the 530 participants, 377 (71.1%) reported no falls, 120 (22.7%) had a single fall, and 33 (6.2%) had recurrent falls. Fallers were generally older, with recurrent fallers being the oldest. Males comprised 54.1% of non-fallers, 40.0% of single fallers, and 42.4% of recurrent fallers. A higher proportion of recurrent fallers (69.7%) and single fallers (55.8%) were physically inactive, compared to 40.9% of non-fallers. Use of mobility assistive devices was more common in recurrent fallers (45.5%) and single fallers (26.7%) than in non-fallers (11.9%). Polypharmacy was present in 63.6% of recurrent fallers, 45.0% of single fallers, and 40.1% of non-fallers.

**Table 1:** Participant characteristics by fall category

Variables	Fall category		
	No fall (n = 377)	Single fall (n = 120)	Recurrent falls (n = 33)
Age (yr)	73.0 (69.0–79.0)	75.0 (70.0–83.5)	77.0 (70.0–81.0)
Body mass index (kg/m^2^)	27.8 (25.2–31.2)	28.1 (24.5–31.0)	30.0 (25.4–32.3)
Sex (male)	204 (54.1)	48 (40.0)	14 (42.4)
Marital status			
Single/never married	12 (3.2)	2 (1.7)	1 (3.0)
Married/relationship	269 (71.3)	70 (58.3)	20 (60.6)
Separated/widowed	96 (25.5)	48 (40.0)	12 (36.4)
Educational status			
No secondary	186 (49.5)	69 (57.5)	20 (60.6)
Secondary	47 (12.5)	14 (11.7)	0 (0.0)
TAFE/trade	60 (16.0)	7 (5.8)	5 (15.1)
University	47 (12.5)	19 (15.8)	6 (18.1)
Post-secondary training	36 (9.5)	11 (9.2)	2 (6.1)
MSPSS score	73 (65.0–82.0)	72 (60.0–80.0)	67.5 (59.5–73.0)
Smoking (yes)	17 (4.5)	4 (3.3)	0 (0.0)
High alcohol consumption (yes)	42 (11.1)	16 (13.3)	2 (6.1)
Sleeping at night (hr)	6.9 ± 1.5	6.9 ± 1.6	6.7 ± 1.7
Mobility (inactive)	154 (40.9)	67 (55.8)	23 (69.7)
MAD use (yes)	45 (11.9)	32 (26.7)	15 (45.5)
Polypharmacy (yes)	151 (40.1)	54 (45.0)	21 (63.6)
Decreased vision (yes)	290 (76.9)	86 (71.7)	21 (63.6)
Charlson comorbidity index	0 (0–1)	0 (1–2)	0 (0–2)
Depression symptoms (yes)	28 (7.4)	17 (14.2)	7 (21.2)
Anxiety symptoms (yes)	53 (14.1)	28 (23.3)	10 (30.3)

Data are presented as median (IQR), mean (± SD) or n (%)

Symptoms of depression and anxiety were assessed using the Hospital Anxiety and Depression Scale. Polypharmacy (≥ 5 medications/day concurrently)

*IQR* interquartile range, *MAD* mobility assistive devices, *MSPSS* modified scale of perceived social support; Recurrent falls ≥ 2 falls, Single fall: one fall, *SD* standard deviation, *TAFE* technical and further education

### QoL domain scores based on fall frequency and pairwise comparisons

**Fig. 1:**
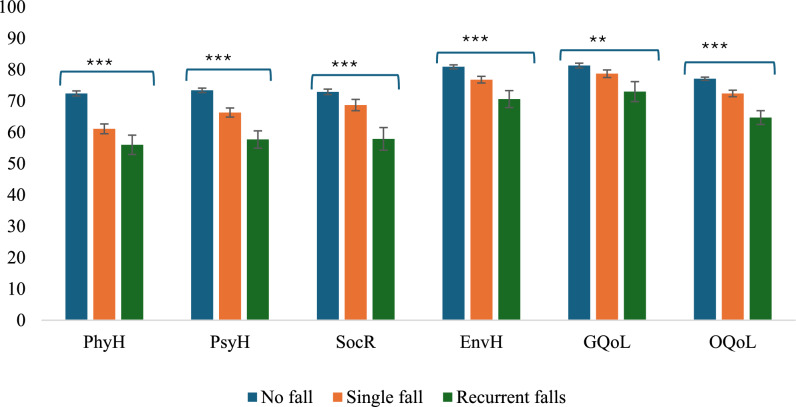
Comparison of QoL domains by fall frequency. Data are presented as mean ± SE. *PhyH* physical health, *PsyH* psychological health, *SocR* social relationships, *EnvH*: Environmental health, *GQoL* global quality of life, *OQoL* overall quality of life, Single fall: One fall; Recurrent falls: ≥ 2 falls; ^***^p < 0.001; ^**^p < 0.05

**Fig. 2:**
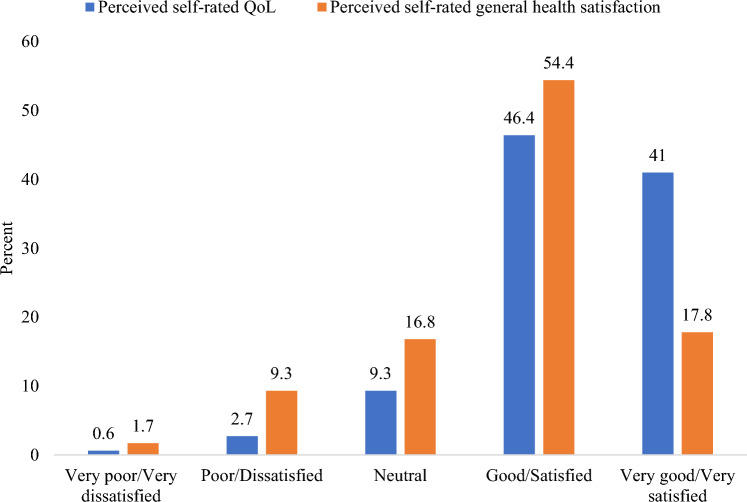
Perceived self-rated QoL and general health satisfaction among participants

QoL scores decreased across physical health, psychological health, social relationships, and environmental health domains as the number of falls increased (Fig. 1). Those with no falls had the highest scores, followed by single fallers, with the lowest scores for those with recurrent falls. All differences were significant. Nearly half (46.4%) rated their overall QoL as good, 41% as very good (Fig. 2), and over half (54.4%) were satisfied with their general health (Fig. 2). For physical health QoL, individuals with a single fall had a significantly lower mean score than non-fallers, with a mean difference of −2.70 points (95% CI −4.41, −0.98). Recurrent fallers also had lower physical health QoL scores than non-fallers, with a mean difference of −3.40 points (95% CI −6.55, −0.24), although the mean difference between recurrent and single fallers was −0.70 points and not statistically significant (95% CI −4.06, 2.65). In terms of psychological health, a graded decline was evident: single fallers had a mean difference of −2.51 points compared to non-fallers (95% CI −4.76, −0.25), and recurrent fallers had a larger mean difference of −5.31 points (95% CI −9.55, −1.08). However, the mean difference between recurrent and single fallers (−2.80 points; 95% CI −7.30, 1.69) was not significant. For social relationship QoL, the mean difference between single fallers and non-fallers was −0.79 points (95% CI −4.82, 3.24). In contrast, recurrent fallers had a lower mean score than non-fallers, with a mean difference of −8.08 points (95% CI −15.23, −0.93), and single fallers (mean difference = −7.29 points; 95% CI −14.76, 0.18). For environmental health QoL, the mean differences were −0.94 (95% CI −3.69, 1.80) for 1 fall vs no falls, −4.52 (95% CI −9.82, −0.78 for 2 + falls vs no falls, and −3.58 (95% CI −9.17, 2.01) for 2 + falls vs 1 fall.

### QoL domains correlation analysis

The correlations between the domains of QoL reveal significant positive relationships. Physical health is moderately correlated with psychological health (r = 0.63), social relationships (r = 0.42), and environmental health (r = 0.52). Psychological health also shows moderate correlations with social relationships (r = 0.54) and environmental health (r = 0.65). Social relationships and environmental health are positively correlated (r = 0.51). Finally, the Global QoL domain shows weak positive correlations with the other domains: Physical health (r = 0.30), psychological health (r = 0.26), social relationships (r = 0.22), and environmental health (r = 0.12) (Supplementary Table 1).

### Falls and QoL domains relationship

During the bivariate analysis, both single and recurrent falls showed a statistically significant association with overall QoL and its specific domains, including physical health, psychological health, social relationships, and environmental health. After adjusting for relevant confounding factors in the multivariable analysis, the study revealed that both single falls (β = −2.35, 95% CI −4.18, −0.52) and recurrent falls (β = −7.53, 95% CI −10.7, −4.32) were associated with a reduction in overall QoL. Recurrent falls related to all QoL domains: physical (β = −5.94, 95% CI −10.8, −1.22), psychological (β = −7.92, 95% CI −13.7, −2.12), social (β = −10.7, 95% CI −18.4, −3.02), and environmental (β = −5.79, 95% CI −11.3, −0.32), while single falls are mainly associated with physical (β = −2.90, 95% CI −5.48, −0.32) and psychological health (β = −3.20, 95% CI −5.59, −0.80) (Table 2).

**Table 2:** Association between falls and QoL domain scores via Tobit regression model

Variable	Bivariable model			Multivariable model		
	β (95% CI)	SE	*P-value*	β (95% CI)	Robust SE	*P-value*
*Overall QoL*						
Falls (Ref = no fall)						
Single fall	−4.68 (−6.86, −2.51)	1.11	< 0.001	−2.35 (−4.18, −0.52)	0.93	0.010
Recurrent falls	−12.4 (−16.1, −8.63)	1.91	< 0.001	−7.53 (−10.7, −4.32)	1.63	< 0.001
*Physical health*						
Falls (Ref = no fall)						
Single fall	−7.31 (−10.7, −3.95)	1.71	< 0.001	−2.90 (−5.48, −0.32)	1.31	0.026
Recurrent falls	−16.4 (−22.2, −10.5)	2.96	< 0.001	−5.94 (−10.8, −1.22)	2.39	0.013
*Psychological health*						
Falls (Ref = no fall)						
Single fall	−7.16 (−10.1, −4.21)	1.50	< 0.001	−3.20 (−5.59, −0.80)	1.22	0.008
Recurrent falls	−15.8 (−20.9, −10.6)	2.60	< 0.001	−7.92 (−13.7, −2.12)	2.95	0.006
*Social relationships*						
Falls (Ref = no fall)						
Single fall	−4.25 (−7.95, −0.56)	1.88	0.024	−1.77 (−4.95, −1.42)	1.62	0.274
Recurrent falls	−15.0 (−21.4, −8.61)	3.26	< 0.001	−10.7 (−18.4, −3.02)	3.92	0.005
*Environmental health*						
Falls (Ref = no fall)						
Single fall	−4.06 (−6.63, −1.48)	1.31	0.002	−1.76 (−4.11, 0.59)	1.19	0.141
Recurrent falls	−10.2 (−14.7, −5.76)	2.78	< 0.001	−5.79 (−11.3, −0.32)	2.78	0.036

*Ref* reference, *CI* confidence interval, *QoL* quality of life, *SE* standard error

The differences between single and recurrent falls were not significant for the physical health (β = 3.30, 95% CI −1.21, 7.82) and psychological health domains (β = −0.44, 95% CI −4.74, 3.86). However, significant differences were observed in the social relationships (β = 6.98, 95% CI 0.10, 13.86) and environmental health domains (β = 4.75, 95% CI 0.12, 9.38), with individuals who experienced recurrent falls associated with a lower QoL than single falls.

## Discussion

Falls are associated with a lower QoL, particularly among older adults. QoL is a key measure of health in older adults [7], and as the population over 65 years grows, there is a greater need to study factors related to QoL, particularly falls, a common geriatric issue, to promote healthy ageing. This study aimed to assess the association between one-year fall history and QoL domains and compare QoL between single and recurrent fallers among older adults in southeastern Australia. The study found that falls were associated with a lower QoL in older adults, with recurrent falls associated with all QoL domains, while single falls were associated with all except social relations and environmental health.

Fallers were associated with 2.4- and 7.5-unit lower overall QoL scores, respectively, compared to non-fallers. This finding is consistent with previous studies, showing that older adults who report falls generally have lower QoL scores than non-fallers. In Korea, fallers had significantly lower QoL, except in the environment domain [17]. Thiem et al. [18] reported QoL reductions of 4.1-units for single falls and 9.0-units for frequent falls, while Boyé et al. [19] found that recurrent fallers had lower scores across all domains. Kantow et al. [36] noted a QoL score drop of about 11-points for those who had fallen in the past year. Interventions like balance training, strength exercises, and home safety modifications are recommended to potentially reduce falls and improve QoL.

Single and recurrent fallers had 2.9- and 5.9-unit lower physical health scores than non-fallers. This finding is consistent with studies conducted in Korea and Norway [17, 20]. Paiva et al. [37] found that fallers had a 9.3-point reduction in physical health scores compared to non-fallers. Older adults who experienced falls had significantly lower levels of physical health [38]. This lower physical QoL is often observed with injuries, pain, and reduced mobility related to falls [9], which can limit their ability to perform daily activities, reduce independence, and diminish overall physical well-being. Boyé et al. [19] found that fallers generally have worse physical health than non-fallers, with recurrent fallers showing even poorer health and QoL than those who fall only once.

Single and recurrent falls were associated with 3.2- and 7.9-unit lower psychological health scores, respectively, compared to no history of falls. This finding is consistent with studies conducted elsewhere [17, 20]. Paiva et al. [37] found that fallers had a 7.5-point reduction in psychological health score compared to non-fallers. Older adults who reported falls had significantly lower levels of mental health [38]. This reduction may reflect increased levels of anxiety and depressive symptoms and fear of falling [3, 20, 39], all of which can adversely influence mental well-being and lead to social withdrawal, decreased self-confidence, and a diminished sense of autonomy. Boyé et al. [19] found that fallers have lower mental health QoL scores than non-fallers, with recurrent fallers experiencing even greater declines than single fallers. Social support and mental health care should be considered to help older adults manage anxiety, depression, and mental health challenges, improving their overall well-being and quality of life [40].

Fallers had lower social relationship scores compared to non-fallers. This finding is consistent with other studies [17]. After a fall, older adults may develop a fear of falling again, leading to social withdrawal, isolation, and reduced engagement with others [41]. Physical injuries from falls can limit mobility, making social participation harder [42]. Recurrent falls can cause depression, anxiety, and increased dependency, all of which reduce social engagement and harm self-esteem. This decline in social connectedness can negatively influence their perceived support, personal relationships, and overall sense of belonging [43], lowering social relationships. Targeted interventions to reduce the frequency and severity of falls can boost older adults' confidence in maintaining and enjoying social relationships, ultimately enhancing their QoL in this domain.

Fallers were associated with lower environmental health scores compared to non-fallers. Falls can impair activities of daily living, limit mobility, and increase dependence on assistance. This restriction influences QoL, particularly in aspects related to safety and environmental accessibility, as shown in studies like that of Adam et al. [44]. The inability to engage with and enjoy one's environment, such as accessing outdoor spaces or completing household tasks [45], negatively influences environmental health. Improving the environmental health domain of QoL can be achieved by addressing fall-related safety concerns through environmental modifications and enhanced mobility support.

Not all falls are equal in their result—people who fall multiple times experience worse outcomes (e.g., lower QoL) than those who fall once [18]. Our findings indicate that although recurrent falls did not worsen physical and psychological health compared to single falls, they were linked to poorer social relationships and environmental health. The observed lower QoL among individuals reporting more frequent falls indicates an association suggestive of an additive impact of recurrent falls.

Although derived from cross-sectional data, these findings highlight a clear association between falls and lower quality of life across multiple domains, revealing important opportunities for intervention. The findings suggest that a holistic approach—including strength and balance training to enhance physical function, social prescribing and community engagement to support psychological and social well-being, and strategies to reduce loneliness and environmental risks—could meaningfully benefit older adults if incorporated into routine care. While causality cannot be confirmed, understanding these associations provides valuable guidance for targeted efforts to preserve and improve quality of life in this population.

### Strengths and limitations

To our knowledge, this is the first study in Australia to assess fall frequency (no, single, recurrent) in relation to each QoL domain using the WHOQOL-BREF. Our findings provide more nuanced insights than previous research, which typically used a simple fall/no-fall classification. Participants were randomly selected from the Australian electoral roll, ensuring representativeness. The study used the reliable and validated Australian version of the WHOQOL-BREF to assess QoL across physical, psychological, social, and environmental domains. While self-reported bias due to relying on participants to recall and report falls [46] may lead to underestimation, particularly for minor falls or those not resulting in injury, our method of identifying falls has previously been shown to be reliable [47]. As this was a secondary analysis, an a priori sample size calculation was not possible. A post hoc power analysis indicated adequate power (87.9%), but given its limitations, findings are best interpreted using effect sizes and confidence intervals. We acknowledge the possibility of reverse causation, whereby lower QoL might predispose individuals to falls rather than being solely a consequence; however, the cross-sectional nature of the data limits our ability to draw causal conclusions.

## Conclusion

This study highlighted the associations between falls and overall QoL among older Australians. Recurrent falls were associated with lower scores across all QoL domains, while single falls were primarily associated with physical and psychological health. Individuals with single falls had higher overall QoL scores compared to those with recurrent falls, but differences were only observed in social relationships and environmental health, indicating that recurrent falls were linked to poorer social and living conditions. A comprehensive approach combining strength and balance training, social support, and environmental modifications can lower fall risk and improve quality of life in high-risk older adults.

## Supplementary Information

Below is the link to the electronic supplementary material.

Supplementary file1 (DOCX 12 kb)

## Data Availability

Data are available upon reasonable request.
