# Supplementary material for: The association between one-year fall history and quality of life among older adults in the Geelong Osteoporosis Study: does fall frequency matter?
Source: Eur Geriatr Med. 2025 Oct 6;17(1):97–106. doi: 10.1007/s41999-025-01324-7 (PMC12945934; doi:10.1007/s41999-025-01324-7)
Supplement: Supplementary file 1 — Supplementary file1 (DOCX 12 kb) [file 41999_2025_1324_MOESM1_ESM.docx]

Supplementary Table 1: Pearson's correlation of overall QoL and its domains.

| Domains of QoL | PhyH | PsyH | SocR | EnvH | GQoL | OQoL |
| --- | --- | --- | --- | --- | --- | --- |
| PhyH | 1 |  |  |  |  |  |
| PsyH | 0.63^**^ | 1 |  |  |  |  |
| SocR | 0.42^**^ | 0.54^**^ | 1 |  |  |  |
| EnvH | 0.52^**^ | 0.65^**^ | 0.51^**^ | 1 |  |  |
| GQoL | 0.30^**^ | 0.26^**^ | 0.22^**^ | 0.12^**^ | 1 |  |
| OQoL | 0.75^**^ | 0.79^**^ | 0.71^**^ | 0.67^**^ | 0.67^**^ | 1 |

PhyH: Physical health; PsyH: Psychological health; SocR: Social relationships; EnvH: Environmental health; GQoL: Global quality of life; OQoL: Overall quality of life; ^**^P<0.001
